# The Relationship between Hunting Methods and the Sex, Age and Body Mass of Wild Boar *Sus scrofa*

**DOI:** 10.3390/ani10122345

**Published:** 2020-12-09

**Authors:** Robert Kamieniarz, Łukasz Jankowiak, Martyna Fratczak, Marek Panek, Janusz Wojtczak, Piotr Tryjanowski

**Affiliations:** 1 Department of Wildlife Management and Forest Protection, Faculty of Forestry and Wood Technology, Poznań University of Life Sciences, Wojska Polskiego 71D, PL-60-625 Poznań, Poland; robert.kamieniarz@up.poznan.pl; 2 Institute of Biology, University of Szczecin, Wąska 13, PL-71-415 Szczecin, Poland; jankowiakl@gmail.com; 3 Institute of Zoology, Poznań, University of Life Sciences, Wojska Polskiego 71C, PL-60-625 Poznań, Poland; martynafrt@gmail.com; 4 Polish Hunting Association, Research Station, 64-020 Czempiń, Poland; m.panek@pzlow.pl; 5 Department of Animal Breeding and Product Quality Assessment, Faculty of Veterinary Medicine and Animal Science, Poznań University of Life Sciences, Złotniki, Słoneczna 1, PL-62-002 Suchy Las, Poland; janusz.wojtczak@interagri.pl; 6 Faculty of Environmental Sciences, Czech University of Life Sciences Prague, Kamýcká 129, 16500 Prague 6, Czech Republic

**Keywords:** conflicts, harvest, hunting, long-term study, wildlife management

## Abstract

**Simple Summary:**

Wild boar *Sus scrofa* is a widely distributed species, the global population of which is continuously growing and its ecological impact is substantial. The increasing number of wild boars results in many conflicts with people, as they cause serious crop damage and are reservoirs of many epidemiologically and economically important diseases. That’s why in many countries to reduce these pressure, hunting for wild boars is carried out. Different methods of hunting and individual preferences of hunters may influence the effectiveness of such population control. We analyzed hunting data from the survey area in Western Poland from the years 1965–2016 and showed that individual hunters and team hunters usually hunt wild boars of different sex and age. Understanding these differences may help future planning of hunting operations, which will ensure an effective reduction of the number of wild boars, without causing untoward changes in the age- and sex structure of the population.

**Abstract:**

Increases in the wild boar *Sus scrofa* population create many conflicts that must be managed, especially because hunting represents a major cause of mortality in this game species. However, hunting effort is not distributed randomly and is influenced by many factors, including hunting methods. This can be especially important in understanding the nature of hunting pressure for both theoretical (ecological and evolutionary) and applied reasons (for management purposes, especially during infectious diseases, for example, African swine fever, outbreaks). We analyzed hunting data from the survey area in Western Poland from the years 1965–2016. In this period a total of 2335 wild boar were culled using two hunting methods: by individual hunters (43.8%) and by teams of hunters (52.0%). During the study period, the number of wild boars increased significantly but in a non-linear manner. More adult males and yearlings of both sexes were shot during individual hunts; more adult females were culled during team hunting. Moreover, the body mass of culled wild boars was positively influenced by the distance to a forest and during the team hunts heavier females and males were shot. To effectively control populations of wild boars, programs to reduce the number of individuals should be better planned and ensure the maintenance of proper age- and sex structure in the wild boar population.

## 1. Introduction

The wild boar (*Sus scrofa*) is a widely distributed species, whose natural range extends from Western Europe and the Mediterranean basin to Eastern Russia, Japan and South-east Asia. As a highly adaptive species, wild boar inhabits semi-arid environments, marshes, forests, alpine grasslands and agricultural land as well as suburban and urban areas [1]. Hunting of this species has a long tradition in Europe and wild boar is valued as a source of meat, bristles and trophies [2,3]. Recently, however, increasing populations of wild boar have resulted in many conflicts between this species and people. Wild boars cause substantial crop damage and are reservoirs of many epidemiologically and economically important pathogens. They can be a source of diseases dangerous to humans, such as hepatitis E, tuberculosis, leptospirosis and trichinellosis, as well as diseases dangerous to domestic animals, such as brucellosis, classical swine fever and African swine fever [4,5]. 

The risk of contracting diseases in humans and livestock is now one of the major motivations for limiting the wild boar population via hunting. This kind of population control started to be especially important after a recent outbreak of African swine fever (ASF) in wild boar and domestic pig populations in Europe [6]. The virus has spread into many European and Asian countries through wild boar, domestic pigs and human activities, threatening the pig meat business and causing serious economic losses [6]. Consequently, many countries have taken drastic measures to control the ASF epidemic. Poland and several other countries have massively increased culling of wild boar to minimize ASF spread and the risk of its transmission to domestic pigs. This decision was taken despite the opposition of many experts in wildlife management [7,8]. 

Effective limitation of the wild boar population cannot be accomplished without population monitoring and understanding of the key drivers of its growth. Only adaptive wildlife management strategies, based on scientific evidence and local circumstances, can lead to successful wild boar population control [9,10]. 

It should be noted, that traditionally wild boars are also hunted for trophies. It has been shown that it is important to understand the harvesting process as a potential source of bias in morphometric measurements and age structure estimates of the local population [11,12,13]. As a consequence, an important part of an adaptive wildlife management approach is understanding what impact hunting methods have on the sex and age of culled individuals, which from a larger perspective can influence the number and demographic structure of the wild boar population. 

Therefore, the present study was established to examine very simple descriptors (body mass, age and sex of wild boars), taking account of inter-seasonal differences, to test for differences among wild boar culled by the following two popular hunting methods: by individual hunting by stalking or posting from the hunting pulpit and team hunting by drive hunts. Obviously, other sources of wild boar mortality occurred in the study area related to natural processes like weather and senescence, as well as anthropogenic ones, especially transport collisions but they were very marginal in comparison to the hunting pressure [2,12]. We further explore the potential of different hunting methods for effective wild boar population management. 

## 2. Materials and Methods 

### 2.1. Study Area

The study was carried out in the years 1965–2016 in the experimental area of the Polish Hunting Association Research Station at Czempiń, western Poland (52°08′ N; 16°44′ E). The initial size of this area was 150 km^2^ but it decreased to 100 km^2^ in 1986 and next increased to 130 km^2^ in 2007. This is a typical farmland region, without fences, thanks to which immigration and emigration of animals is possible. Agricultural land constitutes nearly 70% of the area and cereals are the main crop. Forest patches (40–1300 ha) cover 18% and clumps (<1–15 ha) and strips of trees occupy 3% of the area; the rest of the area (10%) is covered by human infrastructure. The mean annual temperature is c. 8 °C (sub-zero mean monthly temperatures occur in December-February) with mean annual precipitation c. 550 mm. Wild boars locally are given supplementary feeding, especially in winter but sometimes also in summer, to reduce crop damage [14].

### 2.2. Hunting Data

Data on wild boar derive from hunting in the Czempiń area, where wild boars are known to have one litter each year. In Poland, during the period 1965–2016, wild boars were shot throughout the whole year. Age groups were classified as (1) piglets: individuals less than 1 year, (2) yearlings: between 1 and 2 years of age and (3) adults: more than 2 years of age (according to mandibular tooth development) [15]. Hunting in the area was done using two methods: (1) individual hunting by stalking or posting from the hunting pulpit and (2) team hunting by drive hunts. There has been no special preference for either method over the study period nor are we aware of any changes in such pattern over the study period [16]. 

Although individual hunting could be performed all year round, adult females were protected from mid-January to mid-August. Besides, killing leading females during the remainder of the period was generally avoided, as well as small piglets (<20 kg) in summer and fall, although this was not prohibited. Individual hunts took place mainly in forests, on forest edges or among farmland, usually by sneaking or chatting from the ground or platforms. They were implemented by hunters and gamekeepers to obtain meat or a trophy but also to remove wild boars and reduce their numbers to reduce damage in crops. The individual shooting was carried out based on a personal permit, which determined the age group and the number of individuals to be killed in a given area and a specific period. Group hunts were usually held from mid-October to mid-January. They were organized by the manager of the hunting area and consisted in taking out fragments of a forest with the size of 20–100 ha, small field trees and sometimes cornfields and driving animals by battue and dogs towards hunters (from several to 30 people). They could usually shoot without restrictions as to the number and age of wild boars. The number of wild boars shot in this area in a given year resulted from shooting quotas established by local hunting authorities based on previously estimated numbers of wild boar in this area.

### 2.3. Frequency of Hunting and Culling

We analysed the number of culling wild boars using generalised linear models with a Poisson error distribution (performed in R software [17]). The dependent variable was frequency number of wild boars in each category and independent categorical variables were sex (two levels: females and males), age (three levels: piglets, yearlings, adults) and hunting method (two levels: single, team). To analyse differences between groups we included the three-way interaction (sex × age × method) in the model. To test the significance of variable we used the likelihood ratio test (LRT) which compares according to Akaike Information Criterion (AIC) the full model to a reduced model where target variable had been dropped (drop1() function in R software). After finding a significance of the interaction, we compared them by post-hoc Sidak’s tests (package in R software: emmeans [18]). 

### 2.4. Annual Numbers

To model the yearly variation in the number of culled wild boar, we used generalized additive models with a normal error distribution (packages mgcv [19] in R software [17]). Because the size of the hunting area changed over the study years, we standardised the number of wild boar to the area of 100 km^2^ and this variable was used as the dependent variable. 

### 2.5. Body Mass of Culled Wild Boar

We used a general linear model with a normal error distribution (performed in R software [17]); the dependent variable was body mass of culled wild boar. As independent categorical variables, we chose sex, age, hunting method and as a continuous variable, we used distance to a forest from the hunt site (forest distance; if hunting was performed within the forest the value of this was taken as zero), day of the year (doy), year of hunt and the density of culled wild boar per 10 km^2^. We started our analyses by building a model with three-way interactions: age × sex × day of the year, age × sex × hunting method, age × sex × forest distance, age × sex × year. Then, if detected as non-significant (*p* > 0.05) the interactions were removed using a backward elimination method. To test the significance of variable we used the F-test which compares the full model to a reduced model where target variable had been dropped (drop1() function in R software). Multiple comparisons were done with post-hoc Tukey’s test (package emmeans [18]). The final model was diagnosed graphically to check for model assumptions and we did not find any violations. The prediction of the model was drawn using the package: ggplot2 [20] and ggeffects [21].

## 3. Results

### 3.1. Frequency of Culling

In the period 1965–2016 2335 wild boars were reported as being culled (1022 [43.8%] by individual hunters and 1215 [52.0%] by team hunters, 98 [4.2%] no identified; Table S1 in Supplementary Materials). The fitted log-linear model indicates a significant three-way interaction between age, hunting method and sex (df = 2, χ^2^ = 28.486, *p* < 0.001). 

**Figure 1:**
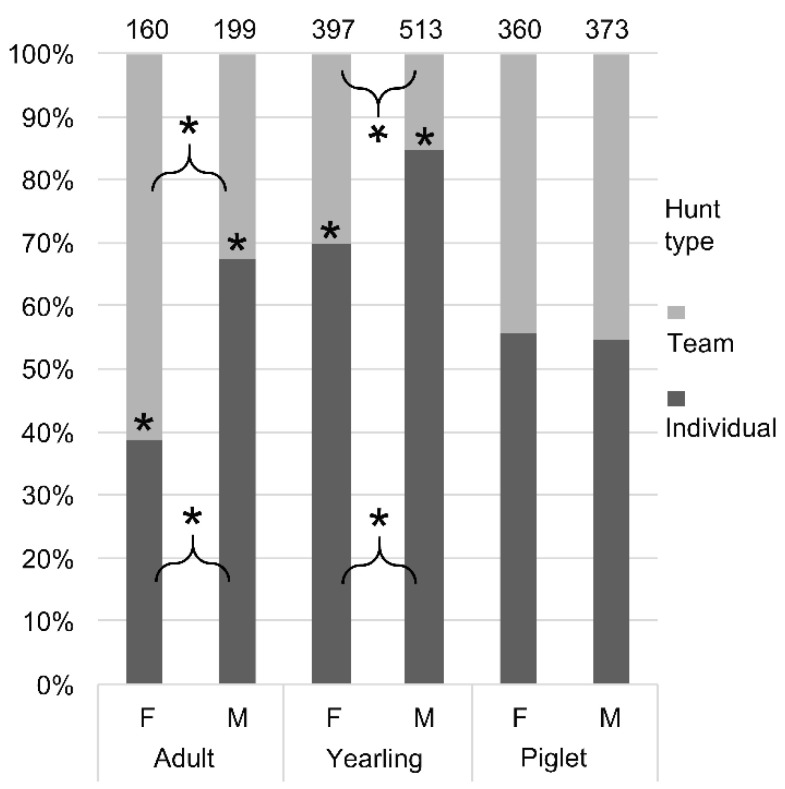
Number of culled wild boar (*Sus scrofa*) in the period 1965–2016 in the Czempiń hunting area in western Poland. The groups (Females [F] vs Males [M] and Individual vs Team hunt type) are compared by post-hoc according to the fitted Poisson model with interaction sex × age × method. * indicate differences at *p* < 0.05.

The results of post-hoc tests are presented in Table S1 in Supplementary Materials. We found that significantly more adult males were shot during individual hunts than during team hunts (Figure 1). The opposite was found for adult females. Male and female yearlings, however, were shot significantly more often during individual hunts than during team hunts. No significant differences were observed for piglets and hunting method.

### 3.2. Annual Numbers

**Figure 2:**
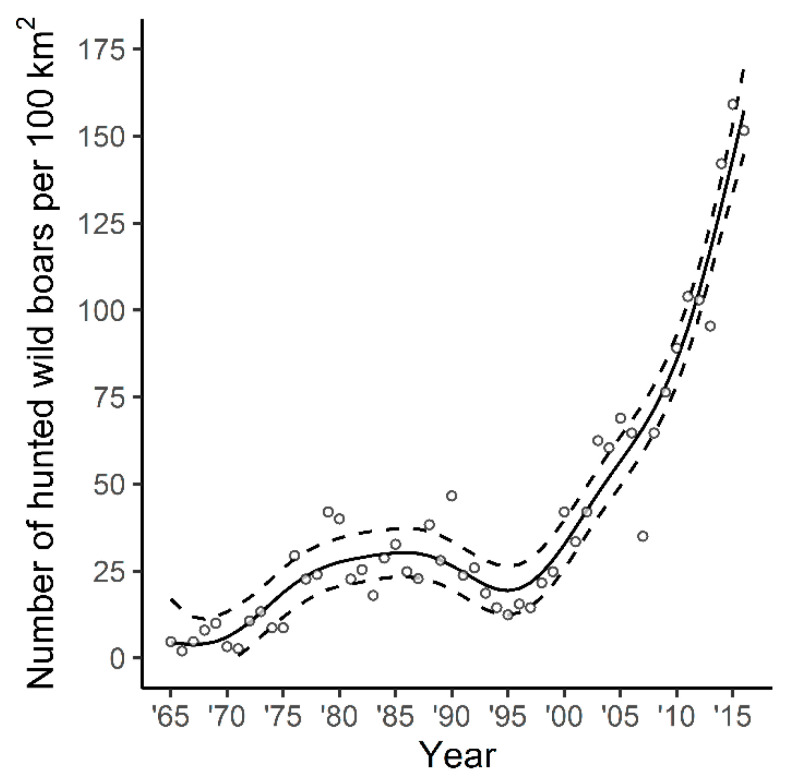
Number of culled wild boar per 100 km^2^ during the study period. The curve is the prediction obtained from the fitted generalised additive model, dashed lines show 95% confidence intervals.

During the study period, the number of culled wild boar increased non-linearly (Figure 2, edf (effective degree of freedom) = 4.5, F = 90.44, *p* < 0.001)—an increase was apparent from 1965 until 1985, followed by a decline during the early 1990s. Since 1998 there has been a large increase in the number of culled wild boar.

### 3.3. Body Mass of Culled Wild Boar

**Figure 3:**
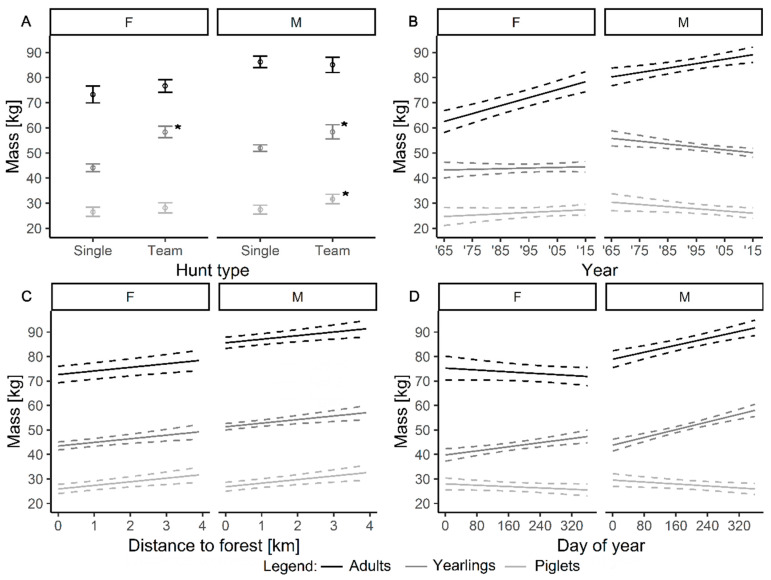
Prediction from the fitted model showing the relationship between different explanatory variables (**A**)—term: age × sex × hunt method; (**B**)—term: sex × year + age × year; (**C**)—term: distance to forest; (**D**)—term: age × sex × day of the year) and mass of culled wild boar (*Sus scrofa*) at the Czempiń hunting area in western Poland. Dashed lines showed 95 confidence intervals. The asterisks in panel A show significant Tukey test comparisons between hunt types (*p* < 0.001). F—females, M—males.

We found the following effects and interactions to be not significant: interaction of age × sex × forest distance (df = 4, F = 0.622, *p* = 0.537), age × forest distance (df = 2, F = 1.970, *p* = 0.140), age × sex × year (df = 2, F = 2.537, *p* = 0.087), number of culled boar per 100 km^2^ (df = 1, F = 0.578, *p* = 0.447). The final best fitted model is shown in Table 1. The model predictions are shown in Figure 3. 

We found interactions of hunting method, sex, age and the mass of culled wild boar. Post-hoc Tukey tests indicated that during the team hunts the mass of culled wild boar was significantly higher for yearling females and males and male piglets (Figure 3A, for each of these comparisons significances at *p* < 0.03) than from individual hunts. 

The mass of culled adult males and females increased during the study period, however, for females, this increase was steeper than for males (*p* < 0.05) (Figure 3B). 

The results revealed a significant effect of distance to forests on the mass of culled individuals: the further the distance to forest, the heavier was culled wild boar. This effect was similar for all age and sex groups (Figure 3C). 

We found differences in the mass of culled wild boar during the season (Figure 3D). During the progress of the season, heavier yearlings and adults were culled in comparison to piglets (both *p* < 0.05). Adult males increase and females decrease their mass during the season (*p* < 0.001).

## 4. Discussion

We found that significantly more adult males were shot during individual hunts than during team hunts, while the opposite was true for adult females. Male and female yearlings were significantly more often culled during individual hunts than during team hunts. No significant difference between numbers of piglets culled during individual hunts and team hunts was found. These results can be explained by different hunt periods of specific categories of wild boar during both individual and team hunts. 

For almost the entire study period, until the end of 2014, adult females were protected from shooting between mid-January and mid-August, hence they were not shot during most of the individual hunting season. In the remaining months, when they could be shot, sows with piglets were avoided by hunters, due to their less than 20 kg body mass and then not profitable for hunters (when considering ratio of shot price vs. meat value) [2,15]. The exact individual to be shot is selected more carefully during individual hunting. Individual hunters shoot mainly at wild boar standing or moving slowly, found in the open - on agricultural areas, on tracks and forest crops or in semi-open mature stands. Individual hunts happen mainly at night, hence it is not possible to shoot in dense parts of the forests. In contrast, team hunting is organized more often in dense forest fragments, which offer more cover for wild boar. Wild boar are driven from their refuges and are often shot crossing narrow forest rides or in gaps in the stand. The hunter focuses on choosing a fixed location to shoot from, hence often has less time to choose a specific individual. Wild boar which first appear within the range of the hunter shot are typically those that get shot. Furthermore, it is easier to hit a larger target, rather than a piglet. Adult males that walk at the end of the group or live in a solitary avoid being killed more often than adult females that are typically guides of wild boar herds [2,3]. Large and heavier males can be preferred during team hunting because some hunting is motivated by the concept of obtaining a trophy [3]. However, the number of culled wild boar of breeding age is limited by the need to distinguish adult males from females and the small proportion of the population in this age group [22]. In the 1990s, the percentage of older wild boars culled in Poland was around 10–11% and in 2007–2008 it increased to 13% [22]. According to hunting management rules recommended in Poland for many years, this percentage should be much higher, around 20% [15,22,23].

Piglets, although they constitute at least half of the population [2], are collected in Poland less frequently than yearlings [22], which is consistent with data in our research. In Poland, it is customary, mainly for economic reasons, to hunt wild boars when they weigh over 20 kg [15]. The economic factor is very important here because managers of hunting areas in Poland allocate income from the sold carcasses of wild boars to pay compensation to farmers for crops damages, caused by game animals [22]. Intensive hunting of yearlings also influence the number of adult wild boars available and, consequently, hunted. 

Yearlings can be hunted throughout the year without having to be distinguished between sexes. As a result, they constituted about half of the wild boars shot in Poland [22] and were the most common age category culled in the study area (44%). Intensive hunting of wild boars takes place especially in spring and summer, because of the need to protect field crops from damage. Another manifestation of the unfavourable culling strategy is the relatively low proportion of adult wild boar.

We found that the number of culled wild boar grew nonlinearly over the period under review—an increase was recorded from 1965 to 1985, followed by stabilization of numbers. This was a consequence of the increasing intensity of hunting during the period of major economic change in Poland. Hunters shot wild boar more intensively because they were charged with the costs of compensation paid to farmers for damage caused to crops. Previously, the compensation to farmers was paid by state [23]. At the beginning of the 1990s, a decrease in the number of wild boars was observed. It could have been a consequence of classical swine fever—causing high mortality among wild boars, which occurred in Poland in the years 1993–1994 [24]. There has subsequently been a large increase in the number of wild boar shot, which is associated with changes in the use of arable land and the intensification of crop production, especially large-scale cultivation and maize cultivation [25].

In the past, the participation of piglets in reproductive activity was rare and occurred in years of abundant masting of oaks or beeches or conditions of intensive feeding by hunters. This was also encouraged by a small proportion of adult wild boar in the population [2,26]. In recent years maize has enriched the environment with a new and valuable source of food, as well as periodic shelter, for wild boar. Thanks to new food sources in a favorable environment, the condition [11] and the reproduction rate of wild boars improved. In the 21st century, the increase in the number of wild boar in Europe was a direct consequence of the increase in the survival rate of young wild boar, larger litters and mating of a much larger number of younger individuals (at <1 year old) than in previous years [27,28,29].

The effect of agricultural land-use changes, ensuring new food sources for animals, could moreover explain the increase of mass of culled adult male and female boar during the study period. On the other hand, the increase in body mass in yearlings and piglets was not observed. These findings could be explained by the earlier reproduction of female wild boar. Younger and thus usually lighter, females produce lighter young [28]. 

An increase of mass during the progress of the season was observed for culled yearlings of both sexes and adult males. This can be explained by wild boar of these categories gaining mass during months of food abundance, also helped by supplementary feeding by humans [13]. In adult females, usually active in reproduction, their mass did not increase during the season. Females participating in reproduction are burdened with lactation in the summer and another pregnancy in the autumn, which now usually begins in October [14]. 

The results revealed also a significant effect of distance to forests on the mass of culled individuals. The further the distance to the forest, the heavier was the culled wild boar. It can be because mainly individuals in good condition explore arable fields, where they may find more food resources and gain higher body mass [25]. This effect reveals a major impact of the structure of the landscape on categories of culled individuals of wild boar. In the future, it will probably result in ecological changes similar to that described for roe deer (*Capreolus capreolus*), which coexist in two subpopulations, of forest and field [30,31]. 

## 5. Conclusions

To conclude, the problems associated with the high number of wild boars, the damage they cause to crops and more recently the emergence of ASF in some countries mean that the main goal of hunting this species is to substantially reduce its population. The pursuit of this goal may, however, impact negatively the structure of the wild boar population. Our research has shown that the number of individuals shot from particular age- and sex groups depends on the hunting method. Specifically, individual hunting is a method that allows selection. However, hunting must be based on relevant legal regulations and takes into account economic considerations. To effectively control the population of wild boars, programs to reduce the number of individuals should be better planned and ensure the maintenance of proper sex-age structure in the wild boar population. For example, the bonus paid for the shooting of wild boar should also apply to piglets, which are reluctantly shot in summer and autumn due to the low carcass weight and thus its value.

## Figures and Tables

**Table 1:** The significance of tested effects on the body mass of culled wild boar. df—degree of freedom.

Terms	df	F-Test	*p*-Value
distance to forest	1	14.18	<0.001
sex × year	1	10.21	0.001
age × year	2	13.89	<0.001
age × sex × doy ^1^	2	8.15	<0.001
age × sex × hunt method	4	3.81	0.004

^1^ a day of the year.

## Supplementary Materials

The following are available online at https://www.mdpi.com/2076-2615/10/12/2345/s1, Table S1: Number of culled wild boar (*Sus scrofa*) in the period 1965–2016 in the Czempiń hunting area in western Poland. The groups (Females vs Males and Individual vs Team hunt type) are compared by post-hoc according to fitted Poisson model with interaction sex × age × method. Comparison and *p* values were adjusted by Sidak’s method.
