# Supplementary material for: The Relationship between Hunting Methods and the Sex, Age and Body Mass of Wild Boar Sus scrofa
Source: Animals (Basel). 2020 Dec 9;10(12):2345. doi: 10.3390/ani10122345 (PMC7764782; doi:10.3390/ani10122345)
Supplement: Supplementary file 1 [file animals-10-02345-s001.pdf]

animals

# Supplementary Materials: The Relationship Between Hunting Methods and the Sex, Age and Body Mass of Wild Boar *Sus scrofa*

**Robert Kamieniarz [1], Łukasz Jankowiak [2], Martyna Fratczak [3], Marek Panek [4], Janusz Wojtczak [5] and Piotr Tryjanowski [3,6,*]**

**Table 1.** Number of culled wild boar (*Sus scrofa*) in the period 1965–2016 in the Czempiń hunting area in western Poland. The groups (Females vs Males and Individual vs Team hunt type) are compared by post-hoc according to fitted Poisson model with interaction sex × age × method. Comparison and *p*-values were adjusted by Sidak's method.

| | | | Hunt type | | | | |
|---|---|---|---|---|---|---|---|
| | | NI[1] | Individual (I) | Team (T) | Total | I vs T OR [2] | *p*-Value |
| Adult | Female (F) | 15 | 62 | 98 | 175 | 0.63 | **0.010** |
| Adult | Male (M) | 24 | 134 | 65 | 223 | 2.06 | **<0.001** |
| Adult | F vs M: OR | | 0.46 | 1.51 | | | |
| Adult | p | | **<0.001** | **0.020** | | | |
| Yearling | NI [1] | 0 | 0 | 8 | 8 | | |
| Yearling | Female | 9 | 277 | 120 | 406 | 2.31 | **<0.001** |
| Yearling | Male | 17 | 435 | 78 | 530 | 5.58 | **<0.001** |
| Yearling | F vs M: OR | | 0.64 | 1.54 | | | |
| Yearling | p | | **<0.001** | **0.006** | | | |
| Piglet | NI [1] | 0 | 1 | 29 | 30 | | |
| Piglet | Female | 33 | 200 | 160 | 393 | 1.25 | 0.070 |
| Piglet | Male | 47 | 203 | 170 | 420 | 1.19 | 0.168 |
| | F vs M: OR | | 0.99 | 0.94 | | | |
| | p | | 0.986 | 0.825 | | | |
| NI [1] | NI [1] | 0 | 14 | 46 | 60 | | |
| NI [1] | Female | 0 | 29 | 19 | 48 | | |
| NI [1] | Male | 0 | 35 | 7 | 42 | | |
| | Total | 98 | 1022 | 1215 | 2335 | | |

[1] not identified (not used in comparisons), [2] odds ratio
